# Suggestions

**Published:** 1875-12

**Authors:** Wm. C. Eastlacke

**Affiliations:** Berlin, Prussia.


### ARTICLE IV.

### *Suggestions.*

BY WM. C. EASTLACKE, D. D. S., BERLIN, PRUSSIA.

[Read before the American Dental Society of Europe.]

We have heard of travelers, visiting for the first time, new lands and new scenes, who, under the enchantment of first impressions, and in the enthusiasm of newly acquired knowledge, have entered into detailed accounts and critical discussions, which would have been widely different, had they waited until the first flush of observation had vanished, and moments of more experienced judgment had toned down their enthusiasm—and yet, in the innocence of their hearts they have imagined that what they did not know was not worth knowing.

Thinking of these, recalls to our minds our "maiden innocency" in the early years of the practice of our Profession: the magnitude of questions then hastily solved, the subtle delicacy of labor then unrealized, in fact, our blissful ignorance of the lights and shades that only twenty years of hard practice and unceasing labor have betrayed.

With each year the love and admiration and enthusiasm for our Profession increases, and yet, it is with a sense of modesty, that we venture to give some details of experience, hoping they may throw some light on our onward course.

Without reference to the favorable or questionable results that may follow the filling of teeth with the various materials now in use, we simply would call your attention to the special cases where *Gold* (the purest of all metals) is employed as the preserver.

A word, first, upon the detection of decay. We need warning in regard to this, for we have so often seen mouths, fresh from the hands of the operator, and judging by the work, the professional brother was skillful in manipulation; accessable cavities were carefully attended to—yet, alas, the patient dismissed with the most important work neglected. We have seen the patient so discouraged, and incredulous too, until we have proved by actual demonstration the existence of decay.

How frequently whilst engaged upon a mouth we are surprised by the discovery of defects, that on first examination we had not suspected. We have had teeth pronounced sound by one operator, reveal to our closer scrutiny a highly decomposed condition.

Now, if we have a right comprehension of our professional responsibility, the first duty is to detect decay, and this is the stepping stone for securing the confidence of our patients—in fact, is the "key stone" to success, and, whether we work for high or low prices, or whether we are, from charitable motives, acting for the good of humanity, the same pure principle should actuate us and the same invariable search be made.

The turned point of a nerve broach should be well used, and when there is any doubt, the operator should not hesi tate in restoring to the revolving disk; so cutting the teeth as not to destroy the contour if possible, and yet better cut than remain in doubt. No harm can be done, but often, in skillful hands, much good can be realized by preventing decay.

We have seen teeth miserably filled by English barbers, long years ago, though unsightly, still good—and the saw-shaped teeth of the fashionable South Sea Islanders are well know for their soundness.

We are not advocating the unnecessary cutting of teeth, and urge that the greatest judgment and skill should be exercised, but are advocating that the search for decay should be made with cautious, but unfaltering perseverance.

The wedge may take the place of the disk and very properly too; wedge and disk are both necessary and safe accessories in skillful and careful hands; and patient observation whilst engaged upon a mouth will often surprise us into new lines of labor, that hurried inattention would never discover.

We have cause to congratulate both ourselves and patient, when our search reveals only perfection.

And now for the preparation of cavities, and here should be the extreme test of skill; for although recent inventions have lessened labor, they only seem to increase the necessity for skillful manipulation and conscientious care.

Not long since we saw it stated in one of the dental periodicals, that "the engine had been the cause of many exposed nerves." With regret we must answer the engine has been the exposure of many bunglers and careless manipulators who are responsible for our Professional honors.

A nicety of touch is indispensable, and not the engine, but the operator is in fault when a pulp is needlessly exposed. In the use of this beautiful assistant, engineering skill is requisite.

Another suggestion may not be misunderstood, viz: Our appointments must not clash—time must be allowed, quick work is well enough when the purse alone is considered, but in cases (which frequently occur) when two or three cavities can be better prepared at one sitting, that is, when better work can be accomplished by thus giving *room* and *light*, then time, undisturbed time, is of the utmost consequence. Although, rather than over weary the patient, one sitting may be given to the preparation of the cavities, and another for the introduction of the gold, the cavities during the interval being filled with creosote and mastic.

Shall we take an example? Notwithstanding, we realize the difficulty in properly demonstrating by words that which would be so much clearer "in hand," but we will endeavor to give you our own "modus operandi" as clearly as possible.

Let us take then for example, the anterior proximal surface of the first superior molar, which is found defective. Also the second bicuspid is found badly decayed on both anterior and posterior surfaces, embracing that portion of the crown between the cusps—and so, correspondingly, is the first bicuspid defective. Examination betrays these cavities as extending beyond the margin of the gum, and so advanced is decomposition, that to make perfect work, we feel the necessity for *seeing clearly all sides of each cavity.* To attain this we begin with the disk and separate well the line of our separation extending from the cervical walls—separating first the second bicuspid and first molar, then the second and first bicuspid, then the first bicuspid and cuspidati. This opens up our work.

The next step is the application of the rubber-dam to the four teeth. Here we have surmounted a difficulty that at first we felt was too discouraging—but gentle persistence has made us expert in its application, and to-day we apply it more readily, and with greater facility, than the napkins of olden time. Now that the rubber is securely adjusted, we can carefully look at the work before us. We excavate then the first bicuspid and can see our way clearly to the anterior proximal cavity in the second bicuspid. Pleasantly surprised with our success, we proceed to excavate the second bicuspid, and with its completion, we have an *unobstructed view* of the anterior cavity in the molar; with what comparative ease is the excavation of this accomplished. In actual practice, we should at this juncture apply the temporary fillings and release our patient, being very willing to postpone the introduction of gold for twenty-four hours at least—but, with your kind indulgence, we will insist on our patient sitting still, and proceed with our work.

Look once again at what is accomplished. Of *every portion of each cavity we have an unobstructed view* and *this* is the advantage we wish to hold, until the conclusion of the operation; therefore we begin first upon the cervical wall of the molar cavity to introduce the gold. Our retaining points have been secured without difficulty, and the little camel hair brush has swept everything clean before us. Our speculum is gently adjusted, so that the cavity appears in the front of the mouth, rather than in the molar.

But to condense. With ease and rapidity we begin upon the cervical wall of the molar cavity; but before building too high, we turn to the cervical wall of the posterior cavity of the second bicuspid, and progress with equal facility, until a firm foundation is secured; then, resuming the filling of the molar, we complete, to our entire satisfaction, and smooth the surface a little so that the gold will not adhere as we proceed with the posterior second bicuspid cavity, which is soon brought along to that part of the cavity lying between the cusps. The anterior cavity is then taken in hand—our work all the time becoming lighter—these two fillings are united by the crown which is then the key that locks each of the others in their places, only the first bicupid remains; we begin on the cervical wall of its posterior cavity and work as smoothly as oil. The result gives us unqualified pleasure, for there has been no hitch anywhere —not a drop of moisture—our patient has actually taken a glass of wine during the operation, and the monotonous tones of the light steel mallet (delicately used by our skillful Japanese,) have put the patient to sleep, whom we awaken with the good news that the work is finished.

In the case that we have had in mind in this statement, the walls of the cavities were so thin we were compelled to cut them away to secure permanency. The question arises, shall we make contour fillings here or not? we know of no other answer, save that this must be left to the judgment of the operator. Contour fillings at times are inadmissible; again, at times, they are necessary; but in the case just described, well-rounded fillings, without any attempt to contour were considered the most advisable, leaving them self-cleansing, that is, with the V shaped separation, which in case of proximal fillings is mostly the best rule. Since the introduction of the rubber dam, the cervical walls, despite large fastoonsof gum, can be so left that food will not crowd between the teeth, thereby rendering the separation objectionable. When such inconvenience occurs we unhesitatingly pronounce it the fault of the artist.

The finishing and polishing of these fillings are so simplified by modern improvements, it would be superfluous to refer to them; and it matters but little as to the gold. It not unfrequently occurs that both light and heavy gold are used in the same cavity; also a thin flooring of oxy-chloride is made before introducing the gold.

Personally, we have for a year or two constantly employed heavy gold, never using under No. 20.

Laminated fillings of heavy foil seem to us the best, (the gold being moderately adhesive,) but this statement is made with all due respect to those of our colleagues who constantly employ light foils, and whose lasting and artistic work is the best evidence in their favor.

The imperfect description that we have just given is the way we treat seven cases out of ten, when the condition of the teeth and patient warrant such a " clean sweep." We know it takes time and scrupulous attention, but the result is most beautiful.

Should the teeth be hyper-sensitive, excavating with the rubber dam facilitates the work, and is a relief to the patient, whilst an application of creosote or carbolic acid with mastic, as temporary filling, has a tendency to reduce the highly sensitive condition of the dentine.

Our work is of too serious a nature to object to the time and care required. Permanent results may be justly anticipated, even though " there is no rule without exception."

And now a word in conclusion in regard to the dread attending dental work. Kindness and forbearance are the indispensable characteristics of a true dentist. An observing physician has an intuitive sympathetic knowledge of his patient, and works accordingly. Even the most nervous will submit without complaint to the removal of the most sensitive dentine by the hands of such an operator, when they could not be induced to enter the office of another.

Our rooms should be so arranged as to take away the suggestion of pain, not making anatomical museums of our offices—exposing instruments, &c. We have no objections to dental museums, but let them be in rooms set apart for the purpose, not where the trembling patient can connect them with anticipated horrors.

We have even sent away our famous dental chair, and operate at a little easy parlor chair, sitting beside our patient on a low light cane-seat stool. Nearly twenty years we stood by improved dental chairs, but find the change we have mentioned far more convenient. Our office presents the appearance of a pleasant sitting-room or library. And the pleased smile of our patients well attests their astonishment and gratitude, whilst that *awful chair* is a remark we never hear.—*Pennsylvania Journal of Dental Science.*

